# Perceptions of Lady Health Workers and their trainers about their curriculum for implementing the interventions identified for Essential Package of Health Services for Pakistan

**DOI:** 10.12669/pjms.37.5.4175

**Published:** 2021

**Authors:** Shaherzad Sohail, Gohar Wajid, Saima Chaudhry

**Affiliations:** 1 Dr. Shaherzad Sohail, MBBS, MCPS, FCPS, MHPE Associate Professor, Gynae/Obs Shalamar medical and Dental College, Lahore, Pakistan; 2 Dr. Gohar Wajid, MBBS, MSC, MPH, PhD Med Education Technical officer, Health Professions Education World Health Organization Cairo, Egypt; 3 Dr. Saima Chaudhry, BDS, MME Officiating Head of Department University of Health Sciences, Lahore, Pakistan

**Keywords:** Universal Health Coverage, Lady Health Workers, Essential Package of Health Services

## Abstract

**Background and Objectives:**

Lady Health Workers (LHWs) form the central cadre of community-based health workers in Pakistan. They must be trained well for implementing community-based interventions identified for Essential Package of Health Services (EPHS) by the government of Pakistan. This study aims to explore the perceptions of LHWs and their trainers about their existing curriculum and identifies gaps in the curriculum for effective implementation of the interventions identified in EPHS.

**Methods:**

In this qualitative study, perceptions of 45 LHWs were taken through focus group discussions and their six trainers were interviewed as well. In addition, three experts analyzed the LHWs training curriculum to identify its relevance with achieving the community-based interventions as described in the EPHS by the government of Pakistan.

**Results:**

Thematic analysis of the information gathered by the interviews of trainers and focus group discussions from LHWs, was performed. Most participants were satisfied with their curriculum but suggested the addition of topics on emerging health issues, neonatal resuscitation, mental health and rehabilitation. Participants felt a deficiency in practical skills, communication skills and leadership skills. Experts identified gap in the current LHWs curriculum to address the recently identified community-based interventions.

**Conclusions:**

The current curriculum of LHWs need reforms to make it compatible with Essential Package of Health Services for Pakistan. The suggested areas for improvement include knowledge of emerging health issues, neonatal resuscitation, adolescent problems, mental health and rehabilitative services.

## INTRODUCTION

Universal Health Coverage (UHC) has become a global priority, and all Member States of the World Health Organization, including Pakistan, have set their targets to make advancements towards it.^1^ UHC implies that all individuals and communities have access to the minimally defined level of health services they need without suffering financial hardship.^2^ However, the journey towards achieving UHC targets has many challenges, the general shortage of well qualified and high quality health workforce being a leading one. Globally, this shortage is expected to increase to 12.9 million by 2035.^3^ The provision of community-based health workers is a practical approach to addressing the shortage of health workers, particularly endorsed by several low and middle-income countries.^4^

Pakistan is a developing country with a limited budget for the ever-increasing health needs of people. The government of Pakistan is striving to expand health services by introducing social health insurance programmes, especially targeting the poor and the most vulnerable groups of the population. The Ministry of National Health Regulations Services and Coordination (MNHRS&C) has developed an Essential Package of Health Services (EPHS) for all populations. Out of 117 identified interventions, 28 will be implemented at the community level by Lady Health Workers (LHWs).

Unfortunately, there is a gross shortage of health workers in Pakistan. Currently, there are only 14.5 doctors, nurses and midwives per 10,000 population in Pakistan,^5^ while WHO suggests a minimum threshold of 44.5 health workers per 10,000 population for achieving SDG targets.^6^ Increasing the density of doctors, nurses and midwives from 14.5 to 44.5 would be a gigantic and costly task and may not even be feasible in a short time. The government needs to explore more cost-effective and practical solutions to combat the shortage of health workers. One solution is to increase the number of community health workers and improve the quality of their training to align them well with UHC targets and 28 interventions identified in the Essential Package of Health Services (EPHS) for Pakistan.

Lady Health Workers (LHWs) are the main cadre of community health workers in Pakistan. Pakistan’s LHW program is recognized as one of the most extensive programs of community health workers in the world.^7^ LHWs are a valuable resource of health sector in Pakistan and form essential workforce for achieving UHC targets by providing primary health care at doorsteps of underserved communities, and slums.^8^ They can accomplish their tasks only if their training is adequate and relevant to the needs of the community. There must be regular enrollment of LHWs to ensure that they cover all population, especially rural and under severed areas. If trained well, LHWs can implement interventions identified in EPHS for Pakistan. There is a need to review their curriculum and align it with the interventions identified in EPHS for Pakistan. We conducted this study to explore the perceptions of LHWs and their trainers regarding the adequacy of their curriculum and identify the gaps in the existing curriculum to align it well with the community-based interventions mentioned in EPHS for Pakistan. This study provides valuable information about the shortcomings in the LHW training curriculum. It will guide the policymakers regarding improvements in a training program to address Pakistan’s future health care needs at Primary Health Care (PHC) level, and in turn, will facilitate the attainment of UHC targets by the country.

## METHODS

A qualitative study was conducted from June 2019 to December 2019 at the University of Health Sciences and Shalamar Hospital, Lahore. Ethical approval (Ref: SMDC/IRB/06-12/135, Dated: Dec 06, 2018) and (UHS/REG-19/ERC/192 dated:11 January 2019) was obtained from the Ethics Committees of Shalamar Medical and Dental College and the University of Health Sciences Lahore. The confidentiality of the participants was maintained at each step of the research. We adopted a case study approach for in-depth exploration of the issue, that is, the training curriculum of LHWs in a real-life context. The perceptions of LHWs and their trainers were explored through focus group discussions, and individual semi-structured interviews regarding their curriculum and suggestions were taken for improvement.

A purposive sampling technique was used to collect data. Lady health workers who had completed their training and serving in the community for at least six months were included in the study. Trainers or teachers who were involved in the training of LHWs were also interviewed. Both LHWs and their trainers had used the standard curriculum approved by the government for their training. After taking the written informed consent from the participants, data were collected through five focus group discussions, each comprising of nine LHWs. Six semi-structured interviews from the trainers were also conducted. The interview questions guide was prepared by the researchers and validated by two experts. All FGDs and interviews were conducted personally by the researcher. These interviews and FGDs were audio-recorded, and notes were also collected during the discussion. The existing curriculum of LHWs was analyzed in detail by three experts, and its relevance with EPHS developed by the government of Pakistan was analyzed. LHW trainers were asked to mark on the proforma prepared by the researcher, which intervention was addressed ‘fully, partially or not at all’ in the curriculum. Data collected was analyzed thematically. After transcribing the data, codes were identified manually based on current issues that emerged from the data. These codes were organized into sub-themes and themes. To ensure the trustworthiness of our research, we ensured meticulous record-keeping and employed member checking. Participants were invited to verify the transcripts. Triangulation of data was also ensured by a thorough review of transcripts by two researchers. For reporting our study, we used Standards for Reporting Qualitative Research(SRQR) guidelines.^9^

## RESULTS

Forty-five LHWs and six trainers participated in five focus group discussions and interviews. About 88% of LHWs have more than five years of work experience and were between 20 to 60 years of age. Teachers had four to ten years of teaching experience. Following six themes and their respective sub themes emerged from the analysis of data. Fig-I

**Fig.1:**
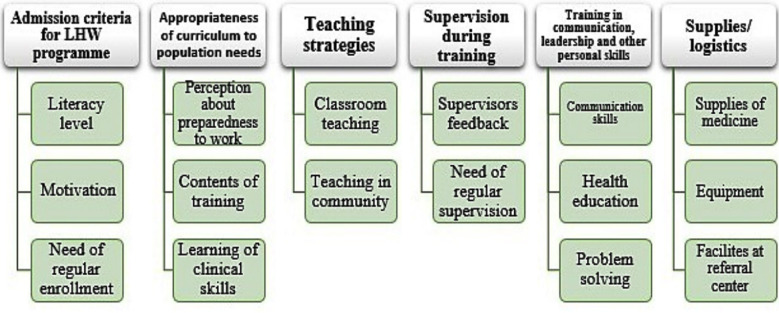
Themes and subthemes emerging from the data.

### Theme 1: Admission criteria for LHWs programme

The trainers emphasized on the selection criteria of LHWs. Although under the current rule the initial schooling of eight years before admission is required, but the trainers felt it must be increased. One trainer suggested:

*“LHWs must have basic qualification preferably up to matric”*.

### Another trainer told

“*Sometimes during selection, the initial literacy level is not followed due to non-availability of the person with the desired level.”*

*“Any tasks like entries and record keeping is not proper if their initial schooling and education are not sufficient.”*

### Theme 2: Appropriateness of curriculum to population needs

Most participants were satisfied with their training and considered the curriculum prepared them adequately to work in the community. The trainers told that training content was reviewed periodically, and new topics were added, like the prevention of AIDs, Malaria and communicable diseases. Participants however stressed that skills based practical training needed revision. Few areas identified included care of newborns, dealing with elderly, taking blood pressure and administering injections etc. Strengthening communication skills was also identified by LHWs that needed to be emphasized.

*“Our main job is to create health awareness, if we can communicate in an effective way only then we can tell our community people how to prevent the disease.”*

### Theme 3: Teaching Strategies

Lady Health workers liked interactive sessions. They learn more while performing role plays, case discussions and demanded more training of practical skills, through simulations and using real setups.

### Theme 4: Supervision during training

Although most LHWs were satisfied with the performance of their trainers, however, participants emphasized that they needed more supervision during ‘on the job training’ in the community. Supervisors also pointed out due to workload at Basic Health Units and lack of transport facility, they were unable to conduct field visit regularly.

### Theme 5: Training in communication, leadership and other personal skills

LHWs pointed out that, as the first point of contact with the community, people had a lot of expectations from with them. One LHW reported,

*“Whenever a new disease or problem spread in the community people expect that we will give them education regarding its prevention and treatment.”*

*“Sometimes we feel difficulty in handling several problems especially when they have a rigid belief about the problem. We need more training in communication with the people.”*

### Theme 6: Logistics and Support

The participants emphasized the need for regular supplies of medicines for common ailments and equipment such as blood pressure apparatus, thermometer and weighing machines. Moreover, LHWs emphasized that diagnostic facilities must be available in the nearby referral centers.

*“People expect that we will provide them with some medicines and they lose the trust of people when we don’t have these medicines.”*

The current curriculum has nine modules which were studied in detail to identify gaps and deficiencies in the curriculum and to align it well with the interventions identified for EPHS for Pakistan. Two experts and the researcher classified interventions into ‘fully covered’, ‘partially covered’ and ‘not covered at all’. Table-I presents the analysis of community-based interventions as identified in Disease Control Priorities-3 (DCP3) based Essential Package of Health Services and their coverage in the existing LHWs curriculum.

**Table-I:** Analysis of Community Based Interventions as identified in Essential Package of Health Services for Pakistan and their presence in the LHWs curriculum.

DCP3 Code	Essential Package of Health Services for Pakistan	Addressed in lady health workers curriculum
C1	Antenatal and post-partum education on family planning	Adequately addressed
C2	Counselling of mothers on providing thermal care for preterm newborn	Partially addressed.
C3a	Management of labor and delivery in low risk women by skilled attendants	Partially addressed
C3b	Basic neonatal resuscitation following delivery	Partially addressed
C4	Promotion of breastfeeding or complementary feeding	Adequately addressed
C5	Tetanus toxoid immunization	Adequately addressed
C8	Detection of severe acute malnutrition and referral	Adequately addressed
C10	Education on hand washing and safe disposal of children’s stool	Adequately addressed
C11	Pneumococcus vaccination	Adequately addressed
C12	Rota virus vaccination	Adequately addressed-
C14	Provision of vitamin A, Zinc and food supplementation	Partially addressed
C16	Childhood vaccination	Partially addressed
C18	Education of school children in oral Health	Partially addressed
C19	Vision screening, vision test	Partially addressed
C27a	Provision of iron and folic acid supplementation to pregnant women and provision of food or caloric supplementation to pregnant women in food insecure households	Adequately addressed
C28	Community based HIV testing with proper referral	Not addressed
C30a	Provision of condoms to key populations	Adequately addressed
C30b	Provision of disposable syringes to people who inject drugs	Not addressed
C32	Contact tracing to identify people exposed to TB	Partially addressed
C43	Early detection and treatment of Chagas disease, Leprosy, Leishmaniasis	Not addressed
C45	Identify and refer patients with high risk pregnancy	Adequately addressed
C46	In emergency infectious outbreak provide advice on how to recognize disease and when to seek medical attention	Adequately addressed
C51	WASH behavior change interventions, such as community led total sanitation	Partially addressed
C53a	Identification/screening of the early childhood development issues motor, sensory and language stimulation	Not addressed
HC4a	Provision of condoms, hormonal contraceptives including emergency contraception	Adequately addressed
HC5a	Counselling of mother on providing Kangroo care for new born	Not addressed
HC9a	Screening of hypertensive disorders in pregnancy	Adequately addressed
HC28	Screening of HIV in all individuals with diagnosis of active TB if present start of HIV care and ARV treatment	Not addressed

Experts identified that out of 28 community-based interventions eight were partially addressed and six interventions were not addressed in the existing LHW curriculum. Table-II summarizes the details of these interventions.

**Table-II:** Summary of interventions identified for Essential Package of Health Services and their relevance with the current Lady Health workers curriculum.

Sr.	Interventions	Number (%)	EPHS Code of intervention
1	Adequately Addressed	13 (46.4)	C1, C4, C5, C8, C10, C11, C12, C27, C30, C45, C46, HC4a, HC9a,
2	Partially Addressed	8 (28.6)	C2, C3a, C14, C16, C18, C19, C32, C51
3	Not Addressed	6 (21.4)	C28, C30b, C43, C53, HC5a, HC28

## DISCUSSION

Most participants of our study realized that their training prepares them well to work in the communities, however a thorough revision of their curriculum is needed to align it with the interventions listed in the EPHS recently introduced by the government of Pakistan. The gaps identified by LHWs and their trainers were related to emerging health issues, mental health disorders, adolescent health problems, neonatal resuscitation and rehabilitative services. A study in Kenya with community health workers also emphasized the need for enhanced training on common diseases and health ethics.^10^ Haq and Hafeez also pointed out the need for continued health education on emerging health issues.^11^

EPHS correctly identified the community’s health needs, and LHWs must be equipped with the skills to address these needs. Another study conducted by Jalal on LHWs in Pakistan highlighted the need for their periodic assessment of training and skills.^12^ The value of the practical training component should not be undermined. Anjum (2016) stressed the need for regular refresher and updating courses of LHWs in Pakistan.^13^

Supervisors play an essential role in the training of LHWs. In our study, LHWs emphasized the need for regular feedback and supervision. A study by Rabbani also emphasized the need for regular feedback.^14^ A systematic review of factors influencing community health workers performance identified direct supervision and continuous training as major factors ensuring better performance of community health workers.^15^

As defined by WHO, UHC ensures everyone, everywhere can access essential quality health services without financial hardships, is one of the top priorities for the government of Pakistan.^16^ The role of LHWs is critical for delivering EPHS to the community and thus achieving UHC targets. DCP3 package identifies 59 interventions involving community platform and can only be effective if community health workers are well trained.^17^ EPHS developed by the government of Pakistan has already identified services to be provided by LHWs.^18^ It is now vital to review the curriculum of LHWs and align it well with the identified interventions. Present research incorporated a detailed review of the LHW curriculum to analyze its alignment with 28 suggested EPHS interventions. Significant gaps in the current curriculum were identified. Only 13 interventions (46.4%) are covered adequately in the existing curriculum. The curriculum needs detailed review to align it well with all interventions.

Preliminary analysis of the curriculum shows that few topics need immediate attention. These include oral health, thermal care of preterm babies, early detection and treatment of Chagas disease, Leprosy, Leishmaniasis. Training regarding rehabilitative services is lacking. Education on emerging health issues, environmental sanitation, hand washing and waste disposal require more emphasis.

EPHS includes new and extended services to be provided at the community level by LHWs like information regarding Kangaroo mother care, a checklist for further evaluation, health education on handwashing, proper disposal of stool, oral health awareness, health education for prevention of STDs, identification and referral of Dengue /Trachoma, information regarding skin infections, adolescent problems, musculoskeletal disorders, wound care and rehabilitative services for Respiratory problems.Table-I Based on the observations from the study, it is recommended that a detailed evaluation of the curriculum be done using program evaluation models (e.g., Kirkpatrick Model) to identify gaps and renew the curriculum to contribute effectively to produce LHWs equipped with new competencies. There is a need to give more emphasis to the skill component in training. Leadership, communication and other personal development skills should be formally developed by making teams and groups during classroom teaching. For those already trained, short term refresher courses can be arranged to upgrade their knowledge, skills and attitudes based on the new competencies.

### Limitations of the Study

We have explored the perceptions of lady health workers and their trainers from one city of Pakistan only. Further studies to examine the views of lady health workers from other areas of Pakistan may be conducted to gain a more holistic picture of the problem. A more detailed expert consultation is required to identify gaps in the existing LHW curriculum and make it more compatible with EPHS interventions identified for Pakistan.

## CONCLUSION

Our research shows that to achieve Universal Health Coverage and SDG targets for health; there is a need for regular recruitment and capacity building of Lady Health Workers. Reforms in their curriculum are needed to make it compatible with the recently developed Essential Package for Health Services for Pakistan. Knowledge of emerging health issues, neonatal resuscitation, adolescent problems, mental health and rehabilitative services needs improvement. Communication skills, decision-making skills and problem-solving skills should be taught formally, and teaching strategies must be made more interactive and student-centered. Supervision during training needs to be increased. The government must plan and implement policies for regular supplies of drugs, equipment and the improvement of facilities in the nearby referral centers.

### Author’s Contribution:

**SS:** Designed the study, collected data, performed and analysis & wrote manuscript.

**GW:** Conceived the idea, contributed to the manuscript, intellectual output and gave final approval

**SC:** Contributed to data collection and analysis and proof reading.
